# Supporting direct acting antiviral medication adherence and treatment completion in a sample of predominantly rural veterans with hepatitis C and substance use disorders

**DOI:** 10.1186/s13722-024-00480-8

**Published:** 2024-06-25

**Authors:** Mary Jane Burton, Andrew C. Voluse, Amee B. Patel

**Affiliations:** 1 G.V. (Sonny) Montgomery VA Medical Center, University of Mississippi Medical Center, 1500 E. Woodrow Wilson Avenue, Jackson, MS 39216 USA; 2 Syneos Health, Morrisville, USA

**Keywords:** Alcoholism, Antiviral agents, Hepatitis C infection, Patient compliance, Substance use disorders, Sustained virologic response

## Abstract

**Background:**

Clinic-based interventions are needed to promote successful direct acting antiviral (DAA) treatment for chronic hepatitis C virus (HCV) infection in patients with substance use disorders (SUDs) among rural Veterans.

**Methods:**

We implemented a clinic-based intervention which used motivational interviewing (MI) techniques to promote medication adherence and treatment completion with 12 weeks of DAA treatment among rural Veterans with chronic HCV and SUDs. Patients received an MI session with a licensed psychologist at baseline and at each two-week follow-up visit during DAA treatment. Patients received $25 per study visit completed. Patients were to attend a laboratory visit 12 weeks after treatment completion to assess for sustained virologic response (SVR).

**Results:**

Of the 20 participants who enrolled, 75% (*n* = 15) completed the planned 12-week course of treatment. Average adherence by pill count was 92% (SD = 3%). Overall SVR was 95% (19/20).

**Conclusions:**

We demonstrated that a clinic-based intervention which incorporated frequent follow up visits and MI techniques was feasible and acceptable to a sample of predominantly rural Veterans with chronic HCV and SUDs.

**Clinical trial registration:**

Registered at ClinicalTrials.gov (NCT 02823457) on July 1, 2016. https://clinicaltrials.gov.

## Background

The incidence of acute hepatitis C virus (HCV) in the United States has more than tripled in the past two decades with increased impact in rural communities as compared to urban ones [1]. This epidemic of hepatitis C infection is fueled by a concurrent epidemic of substance use disorders (SUDs), in particular opioid use disorder in predominantly rural settings [1, 2]. Although HCV is easily cured with a brief and well-tolerated course of direct acting antivirals (DAAs), HCV treatment rates remain low among patients with SUDs [3]. Barriers to HCV treatment among patients with SUDs include limited access to subspecialty care which is amplified by healthcare shortages in rural areas [4] and provider misperceptions of reduced treatment efficacy in this subgroup [5]. Although SUDs were a risk factor for DAA treatment failure in an human immunodeficiency virus (HIV)/HCV cohort [6], other studies have demonstrated that with adequate support patients with SUDs can achieve sustained virologic response (SVR) rates equivocal to those without SUDs [7, 8]. The American Association for the Study of Liver Disease/Infectious Diseases Society of America HCV treatment guidelines support prescription of DAAs among patients with SUDs, particularly in settings with multidisciplinary care which address SUDs and other comorbidities [9].

Reported medication adherence and treatment completion rates to DAAs among SUDs cohorts vary, likely because of the setting and degree of support provided. A highly structured clinical trial conducted in a medication for opioid use disorder (MOUD) treatment center reported DAA medication adherence rates exceeding 95% of doses and a treatment completion rate of 97% [8]. In comparison, a real-world study among urban MOUD centers which offered adherence support reported a DAA medication adherence rate of 78% and a treatment completion rate of 86% [10], while a government supported primary care center for people who inject drugs in Australia reported a 68% DAA treatment completion rate [11]. As access to specialty care for SUDs is suboptimal in rural areas of the US [2], easily duplicated care models which promote medication assistance and HCV treatment completion in rural clinics are needed. The National Viral Hepatitis Action Plan also supports multidisciplinary approaches to HCV treatment among patients with SUDs and calls for research into practices that promote medication adherence and treatment completion [12].

We theorized a clinic-based intervention which combined frequent follow-up visits (every two weeks) with motivational interviewing (MI) techniques could promote medication adherence and treatment completion for rural Veterans with SUDs. While MI was initially developed to promote abstinence in those with alcohol use disorders [13], in recent years adaptations of MI have been applied to the management of chronic medical conditions, including HIV [14]. Randomized controlled studies have demonstrated that MI can promote medication adherence among patients with HIV infection [15]. Core MI techniques have also been applied to improve clinic attendance and retention in care among HIV patients with documented nonadherence [16]. Less is known, however, about the effects of MI on HCV medication adherence or treatment completion. One prior study has associated self-efficacy (defined as one’s perception of their ability to execute a specific behavior or outcome), an idea conceptually linked to MI, with the reduced risk of missing HCV medication doses [17]. The purpose of this manuscript is to describe the implementation of a clinic-based intervention aimed at increasing DAA medication adherence and treatment completion among rural Veterans with HCV and SUDs.

## Methods

### Setting

The G.V. (Sonny) Montgomery VA Medical Center (GVSMVAMC) is an academically affiliated medical center in Jackson, Mississippi. GVSMVAMC provides care to approximately 39,000 unique Veterans annually, many of whom reside in rural areas. Over 80% of counties in Mississippi are designated as rural or highly rural [18]. The hepatitis clinics in the facility meet three days weekly and, to date, have provided DAAs to over 600 Veterans with chronic HCV. In accordance with VA policy, the clinics do not require a period of abstinence from alcohol or illicit substances before initiating DAAs.

### Screening

Patients were screened for participation during a regular hepatitis clinic visit. Inclusion criteria consisted of current SUDs (defined by an ICD code within the past six months and/or baseline Alcohol Use Disorder Identification Test [AUDIT] ≥ 8 or Drug Abuse Screening Test [DAST] ≥ 3) and treatment-naïve genotype (GT) 1 chronic hepatitis C. Patients with GT 1a infection were required to undergo baseline nonstructural protein 5a resistance associated substitution (NS5A RAS) testing (HCV GenoSure™ NS5A; Monogram Bioscience). Patients were extensively counseled about risk factors of HCV and factors associated with liver disease progression. Referral for SUDs treatment was encouraged by the intervention team but not required for participation. SUDs treatment was defined as receipt of MOUD and/or participation in outpatient psychotherapy for SUDs or in a residential SUDs treatment program. Exclusion criteria included acute HCV, decompensated cirrhosis, contraindications to elbasvir/grazoprevir, and an inability to provide informed consent. Females were excluded if pregnant, nursing, and/or unwilling to use contraception. Male sex partners of females who were pregnant, nursing, and/or unwilling to use contraception were also excluded. Participants who met inclusion criteria and agreed to participate provided written informed consent. The study received approval from the GVSMVAMC’s Institutional Review Board.

### Measures

The Visual Analog Scale (VAS) is an easily administered, single-item self-report measure that was selected to assess medication adherence in this study as it has been found to be reliable and valid in an HCV population [19]. A pill count was also used to assess medication adherence.

### Baseline visit

Patients completed a survey of demographic and medical information. Distance traveled to medical center in miles was self-reported. Patients reported address was used to classify residence as rural or non-rural as defined by Health Resources and Services Administration Rural-Urban Commuting Area (RUCA) codes [20]. Chart reviews were conducted for baseline medical and psychological comorbid conditions, as defined by ICD code within the past six months. Patients were prescribed elbasvir/grazoprevir according to the package insert and sent to the on-site pharmacy for same day pick up. Patients were given the date and time of their next appointment and contact information was verified before leaving the clinic. Medications were provided by Merck (IIS 53,635) and were dispensed at the on-site pharmacy at two-week intervals. Clinic attendance was not required to obtain DAA refills.

### Follow-up visits

Follow-up visits occurred at two-week intervals (Weeks 2, 4, 6, 8, 10 and 12) until the medication course was completed. The two-week interval follow-up was chosen to reinforce medication adherence and has been used in HCV studies in similar populations [8]. As this pre-COVID era study was conducted in an institution that did not have a strong telehealth infrastructure, telephone or video delivery was not a viable option at the time. Due to the rural setting (and having to travel long distances for service), office visits with the hepatitis and SUDs clinics were combined, as both were housed in the same building. A laboratory appointment was scheduled 12 weeks after medication completement to assess for SVR-12. A written schedule of appointments, including the SVR-12 visit, was provided. Side effects and tolerance of medications were monitored at each follow-up visit. A single question was used to assess for any substance use since the last visit, with quantity and frequency being recorded if the response was positive.

DAA medication adherence was assessed at each follow-up visit using the VAS and pill counts as previously described [19]. Routine laboratory monitoring occurred every four weeks and included a CBC, CHEM 14, HCV RNA, and a urine sample analyzed for cocaine, marijuana, opioid, and amphetamine use. Patients were provided $25 for each study visit they attended. If a patient did not attend the clinic appointment, they were contacted multiple times by phone and mail to reschedule and/or pick up medication refills.

### Intervention

Patients participated in a 60-minute session with a licensed psychologist with extensive knowledge and experience in MI (author AV) at Weeks 0 and 2 and four 15-minute sessions at Weeks 4, 6, 8, and 10. The sessions at Weeks 0 and 2 focused on clarifying values and creating short-term goals within those values. The therapeutic approach of all sessions was founded in an MI interaction style and served to establish and maintain rapport, elicit change-oriented talk and increase self-efficacy, select a specific target behavior to change, and plan specific practical steps for making such changes. Specifically, during the first two sessions values were elicited that were directly (e.g., “My health is important to me”) and indirectly (e.g., “My family is important to me, and I need to be healthy to have time for my family”) related to health behaviors and medication adherence. Veterans reflected on how well they are living in accordance with their stated values and identified specific goals and objectives for increasing valued living using goal-setting exercises. The sessions in Weeks 4, 6, 8, and 10 were predominately brief “check-in” meetings focused on helping participants problem solve barriers to moving towards their values and reaching identified goals. The intervention was manualized, with each session having distinct but connected topics, goals, in-session exercises, and corresponding homework assignments.

### Data analysis

The primary outcome of interest was the percentage of patients completing 12 weeks of treatment, which was defined as attendance at the last treatment visit (i.e., Week 12). A secondary aim was to evaluate medication adherence to DAA therapy in this population using the VAS and pill count. Chi-square tests of independence were used to compare two dichotomous variables, substituting Fisher’s exact test for instances of expected cell counts less than five. Continuous variables were compared using Student’s *t*-tests. Data was analyzed using SPSS Statistics (IBM). *P* values ≤ 0.05 were considered significant.

## Results

### Screening

Between April 2017 and December 2019, 37 patients were screened for enrollment, of which 20 enrolled. Patients who declined participation were more likely to be younger (57.8 years old [SD = 12 years] vs. 64.2 years old [SD = 6 years], *p* = 0.02) and white (53% vs. 85%, *p* = 0.03). The most common reason for not participating was apprehension of clinical research (*n* = 10; 58%). Two patients (12%) preferred a different treatment regimen and two (12%) cited inability to keep follow-up appointments. Three patients (18%) were deemed ineligible for study participation because of critical drug-drug interactions (*n* = 2) or inability to provide informed consent (*n* = 1).

### Study population

All study participants were male, and most were black (17/20, 85%, Table 1). Most patients (17/20, 85%) had GT 1a infection with no baseline NS5A RAS to elbasvir; 15% (3/20) had GT 1b, thus all received 12 weeks of DAA treatment. Six patients (30%) were actively engaged with SUDs treatment services during the study. One patient (5%) was prescribed MOUD. Five patients (25%) were participants in a residential SUDs treatment program; the average overlap in residential treatment and study participation was 20.8 (SD = 6.3) days.

**Table 1:** Baseline characteristics of study participants (*n* = 20)

	# (%)
Age, median, years (IQR)	65 (6.0)
Black race	17 (85)
Distance traveled to appointments, median miles (IQR)	41 (60)
SUD treatment engagement	6 (30)
AUDIT score^1^, mean (SD)	12.4 (8.7)
DAST score^2^, mean (SD) (*n* = 16)	4.6 (3.5)
SUD diagnoses^3^	
Alcohol	16 (80)
Cannabis	12 (60)
Cocaine	10 (50)
Opioid	5 (25)
Psychiatric diagnoses^3^	
Depressive disorder	8 (40)
Anxiety disorder	7 (35)
Post-traumatic stress disorder	3 (15)
Schizophrenia	3 (15)
Bipolar disorder	2 (10)
HCV Genotype	
1a^4^	17 (85)
1b	3 (15)
ALT, median, IU/L (IQR)	61.5 (31.5)
FIB-4 score, median (IQR)	2.0 (1.0)
HCV RNA, median, log IU/mL (IQR)	6.21 (0.62)

IQR-interquartile range, SD-standard deviation, SUD-substance use disorder, ALT-alanine aminotransferase, IU-international unit, L-liter, FIB-4 Fibrosis-4, HCV-hepatitis C virus, mL-milliliter

^1^ Alcohol Use Disorders Identification Test, performed on all patients

^2^ Drug Abuse Screening Test, performed on patients with ICD code past 6 months for illicit drug use

^3^ Defined by ICD code past 6 months, participants could contribute > 1 diagnoses

^4^ No evidence of NS5A RAVs at position(s) 28, 30, 31 or 93, HCV GenoSure™ NS5A (Monogram Biosciences)

No patients were homeless or inadequately housed. Most patients (17/20, 85%) resided in a rural area as defined by RUCA codes. In addition to SUDs diagnoses, 55% (*n* = 11) were diagnosed with another mental health conditions; the most common psychiatric co-morbidity being depressive disorder (*n* = 8, 40%). Observed medical conditions included essential hypertension, dyslipidemia, and benign prostatic hypertrophy (*n* = 5, 40% each diagnosis). No enrolled patient had cirrhosis, HIV, or chronic hepatitis B.

### Medication adherence, treatment completion and SVR-12

Average adherence by pill count was 92% (SD = 3%) and average adherence by VAS was 93% (SD = 2%). No significant differences were observed in average level of adherence determined by VAS as compared to pill count (*p =* 0.39).

Fifteen patients (75%) completed all study visits. The average length of treatment completion was 10.8 ± 2.6 weeks. SVR-12 was confirmed for 19 of the 20 participants (95%). All 15 patients who completed study visits attended the SVR-12 laboratory visit as scheduled and all achieved SVR-12. Of the five patients who did not complete treatment, only one (20%) attended the scheduled SVR-12 laboratory visit; this patient experienced virologic failure. The remaining four (80%) were contacted by telephone and/or letter at the time of the missed laboratory visit and every three months until SVR was assessed. Two patients were assessed for SVR at 6 and 20 weeks after the original SVR-12 date. The remaining two patients were assessed while hospitalized at 8 and 105 weeks after the original SVR-12 date.

Most patients (16/20, 80%) continued to use alcohol and/or illicit drugs while on DAA therapy; 60% (*n* = 12) reported continued alcohol use, 60% (*n* = 12) reported continued marijuana use, and 40% (*n* = 8) reported continued cocaine use. Continued alcohol, marijuana, or cocaine use was not associated with lack of treatment completion or lack of SVR (all *p* > 0.05).

## Discussion

Treatment of patients with SUDs is essential for reducing HCV as a public health threat. Modeling studies demonstrate that providing DAAs to patients with SUDs and HCV is cost-effective and prevents new infections [21, 22]. Treatment uptake among patients with SUDs and HCV remains low, particularly in rural, medically underserved areas of the United States. Models of care which promote DAA adherence and treatment completions in these populations are needed. We demonstrated the feasibility and acceptability of a structured clinic-based intervention among a predominantly rural sample of Veterans with HCV and concurrent SUDs.

Adherence to DAAs was high in our sample, consistent with prospective, real-world observations [23]. The rates of treatment completion and SVR in our sample mirrors the results observed in structured clinical trials of DAA therapy in urban MOUD treatment centers [8, 10], suggesting clinic protocols which include MI may promote successful DAA therapy in rural patients with SUDs. We did not observe a relationship between ongoing alcohol, marijuana, or cocaine use and treatment failure, which confirms that a period of abstinence should not be a pre-requisite for DAA prescription.

Post-treatment lost-to-follow-up is a known challenge for the comorbid HCV and SUDs population [11]. Although we eventually assessed all patients for SVR, one-fifth of our cohort required additional time and effort to obtain this assessment. We have several suggestions that could improve retention in care for this population. First, our intervention period did not extend beyond the length of medication therapy and may have contributed to loss to follow up. We recommend that future efforts include the time between medication completion and SVR assessment. Secondly, a 12-week DAA regimen was used for patients enrolled in this study; briefer courses of DAA therapy are available [9] and could promote further increases in treatment completion among rural patients with SUDs. Finally, telemedicine strategies may also promote retention in care among rural patients with HCV and SUDs [24].

Limitations to our study include a single-center design, small sample size, and lack of control group. A potential confounding variable includes the addition of incentive pay for participation, although this is theoretically consistent with contingency management, a well-established and validated practice [25]. As a study of Veterans with HCV and SUDs, our findings may not be generalizable to other groups.

## Conclusions

We implemented a clinic protocol which included MI to promote treatment completion among a predominantly rural sample of Veterans with HCV infection and SUDs. Treatment completion and SVR rates were similar to more rigorous interventions among cohorts of urban patients with HCV and comorbid SUDs. We conclude that clinic-based interventions which include MI techniques are feasible and acceptable among rural Veterans with chronic HCV and SUDs.

## Data Availability

The data that support the findings of this study are available on request from the corresponding author [MJB]. The data are not publicly available as they contain information that could compromise research participant privacy/consent.

## Abbreviations

AUDIT: Alcohol Use Disorders Identification Test
DAA: Direct Acting Antiviral
GVSMVAMC: G.V. (Sonny) Montgomery VA Medical Center
HCV: Hepatitis C Virus
HIV: Human Immunodeficiency Virus
MI: Motivational Interviewing
MOUD: Medication for Opioid Use Disorder
RUCA: Rural-Urban Commuting Area Codes
SUDs: Substance Use Disorders
SVR: Sustained Virologic Response
VAS: Visual Analog Scale
